# MEF2C and miR-194-5p: New Players in Triple Negative Breast Cancer Tumorigenesis

**DOI:** 10.3390/ijms241814297

**Published:** 2023-09-19

**Authors:** Sara Caetano, Ana Rita Garcia, Inês Figueira, Maria Alexandra Brito

**Affiliations:** 1 iMed—Research Institute for Medicines, Faculty of Pharmacy, Universidade de Lisboa, Av. Prof. Gama Pinto, 1649-003 Lisbon, Portugal; sara.caetano1@campus.ul.pt (S.C.); arcgarcia@edu.ulisboa.pt (A.R.G.); ines.figueira88@gmail.com (I.F.); 2 Department of Pharmaceutical Sciences and Medicines, Faculty of Pharmacy, Universidade de Lisboa, Av. Prof. Gama Pinto, 1649-003 Lisbon, Portugal; 3 Farm-ID—Faculty of Pharmacy Research and Development Association, Av. Prof. Gama Pinto, 1649-003 Lisbon, Portugal

**Keywords:** cytokeratin, epithelial–mesenchymal transition, invasiveness, MEF2C, migration, miR-194-5p, triple negative breast cancer, tumor suppression, tumorigenesis, vimentin

## Abstract

Among breast cancer (BC) subtypes, the most aggressive is triple negative BC (TNBC), which is prone to metastasis. We previously found that microRNA (miR)-194-5p is downregulated at the early stages of TNBC brain metastasis development. Additionally, the transcription factor myocyte enhancer factor 2 (MEF2)C, a bioinformatically predicted miR-194-5p target, was increasingly expressed throughout TNBC brain metastasis formation and disease severity. However, the contributions of these two players to malignant cells’ features remain undetermined. This study aimed at disclosing the role of miR-194-5p and MEF2C in TNBC tumorigenesis. The transfection of 4T1 cells with a silencer for MEF2C or with a pre-miRNA for miR-194-5p was employed to study TNBC cells’ phenotypic alterations regarding epithelial and mesenchymal markers, as well as migratory capability alterations. MEF2C-silenced cells presented a decline in both vimentin and cytokeratin expression, whereas the overexpression of miR-194-5p promoted an increase in cytokeratin and a reduction in vimentin, reflecting the acquisition of an epithelial phenotype. Both treatments reduced TNBC cells’ migration. These results suggest that MEF2C may determine TNBC cells’ invasive properties by partially determining the occurrence of epithelial–mesenchymal transition, while the overexpression of miR-194-5p promotes a decline in TNBC cells’ aggressive behavior and reinforces this miRNA’s role as a tumor suppressor in TNBC.

## 1. Introduction

In 2020, breast cancer (BC) became the type of malignancy with the most diagnosed new cases per year, ranking fifth as a leading cause of cancer-related death worldwide [1]. Due to its high metastatic potential within the first 3 to 5 years, triple negative BC (TNBC) is the most aggressive and has the worst survival rate of all BC subtypes, with an incidence of around 20% [2,3,4,5]. In BC patients, the risk of distant recurrence is higher in the first decade after diagnosis, with TNBC mainly metastasizing to the brain and lungs [6]. Overall, metastases are responsible for 90% of all BC deaths, making metastatic BC, particularly metastatic TNBC, the leading cause of death in BC patients [6,7]. This aggressive behavior, coupled with the fact that the most commonly used therapies have low response rates in patients with the metastatic form of BC [5], highlights the need for new, effective, and long-term therapeutic approaches. To this end, disclosing the players involved in tumorigenesis appears crucial to reveal novel potential therapeutic targets.

Metastasization from a primary tumor to the ‘target organ’ is a complex multistep process, known as the metastatic cascade, which comprises the local invasion of the neighboring normal tissue; intravasation into circulation; survival in circulation and arrest in a distant organ site (homing); extravasation to the new organ; and colonization [3,8]. The metastatic cascade starts with BC cells undergoing phenotypic and morphological alterations, known as epithelial–mesenchymal transition (EMT) [3]. This reversible process increases cell invasiveness, since tumor cells acquire a migratory and mesenchymal phenotype, concomitant with the loss of cell–cell junctions and a reduction in epithelial cell markers [9,10,11]. During EMT, not all epithelial trades are lost, which gives tumor cells the ability to form clusters, allowing them to invade and migrate together [3,8]. Following EMT, malignant cells detach from the original site of the tumor and travel towards the surrounding tissue (stroma), until they reach a blood vessel [9]. Upon contact with the capillary endothelium, BC cells can enter the circulation, a process known as intravasation [3]. Once in the target organ, tumor cells undergo the reverse process of EMT, known as mesenchymal–epithelial transition (MET) [3]. MET is characterized by the loss of the previously obtained mesenchymal markers and the acquisition of epithelial markers. Despite such phenotypical alterations, a partial MET in BC cells after reaching the brain has been observed, suggesting that these cells retain some migratory properties, which allows them to further disseminate to other areas [12].

Among the myocyte enhancer factor 2 (MEF2) family of transcription factors is MEF2C. This transcription factor plays a key role in many developmental processes and its deregulation affects cell differentiation [13,14]. This ultimately leads to an increase in cellular proliferation, which is of particular interest in cancer [14,15]. MEF2C’s role in tumor development presents some duality accordingly with its subcellular location. In fact, in hepatocellular carcinoma, cytoplasmatic MEF2C inhibited malignant cell proliferation, whereas nuclear MEF2C was associated with increased malignancy through angiogenesis and tumor invasion [16]. Interestingly, MEF2C was shown to have an oncogenic role in colorectal cancer, myeloid leukemia, and pancreatic ductal adenocarcinoma, where its upregulation was associated with disease progression [17,18,19]. However, the biological role played by MEF2C in BC, and particularly in TNBC, is still not fully understood. Previous studies from our team [20] showed that MEF2C is expressed in perivascular BC cells, in the early stages of metastatic development, as well as in well-established metastases, in a mouse model of BC brain metastases (BCBM) formation. This study further revealed MEF2C’s increasing tendency to translocate from the cytoplasm to the nucleus during BCBM development [20], in line with the fact that MEF2C is a transcription factor. Our additional studies in resected brain metastases from BC patients [21] validated the observations in mice and further revealed MEF2C’s different subcellular localizations. Interestingly, the presence of MEF2C nuclear staining in BC cells was associated with an increased number of metastases and larger tumor size, suggestive of a link between MEF2C’s translocation and disease severity. By directly interacting with effector proteins, MEF2C plays a crucial role as a regulator in several signaling pathways, such as Ca^2+^, mitogen-activated protein (MAP) kinase, Wnt, and phosphatidylinositol 3-kinase (PI3K)/protein kinase B (Akt) [15,22]. As these pathways have been reported to be altered in BC tumorigenesis [23,24,25,26,27], including in BCBM formation [21], MEF2C appears as a new player in BC, remaining undetermined its contribution to tumorigenesis.

Recently, microRNAs (miRNAs or miR-), small and highly conserved non-coding RNA molecules that have essential roles in many biological processes, have been highlighted as possible biomarkers in cancer [28,29,30,31]. miRNAs can function both as tumor suppressors and as oncogenes [32], and the same miRNA can present such duality of effects depending on the cell and cancer type [33]. miR-194-5p is an example of this duality, as it has been reported to act as an oncogene in BC tissues [34] and in prostate cancer [35], where its overexpression was positively related with metastatic development and a poor prognosis [35,36,37,38]. On the other hand, it was described to function as a tumor suppressor in glioblastoma [39] and in non-small-cell lung cancer [40], where its downregulation increased the proliferative profile of cancer cells and was indicative of a poorer survival rate [39,40]. In accordance, our previous work [20] showed miR-194-5p downregulation in plasma prior to BCBM development in a mouse model. These findings were further validated in brain sections, where a decreased number of miR-194-5p-positive cells was observed in mice developing BCBM as compared with control animals [41]. Moreover, in an in vitro model of BCBM formation, miR-194-5p and miR-205-5p appeared to be expressed and released by BC cells, by endothelial cells and during their interaction [42]. Altogether, the downregulation of miR-194-5p seen both in vivo and in vitro points to this miRNA as a putative biomarker of the occurrence of brain metastases in BC, and a possible therapeutic target to be explored. Interestingly, a bioinformatics analysis revealed MEF2C as a target of miR-194-5p [20], raising the interest in further exploring the effects of this transcription factor and of this particular miRNA in BC cells’ behavior.

In this work, we aimed at disclosing the role of miR-194-5p and MEF2C expression in TNBC tumorigenesis. The study of phenotypic alterations revealed that MEF2C-silenced cells presented a decline in both vimentin and cytokeratin expression, whereas the overexpression of miR-194-5p promoted a reduction in vimentin and an increase in cytokeratin. Moreover, both treatments were associated with a loss of migratory capability in TNBC cells. Collectively, these results point to the partial occurrence of EMT in TNBC cells, determined by MEF2C, while the overexpression of miR-194-5p resulted in a decline in TNBC cells’ aggressive behavior and reinforces this miRNA’s role as a tumor suppressor in TNBC. By revealing miR-194-5p and MEF2C’s contributions to tumorigenesis, this study discloses novel modulation targets in TNBC that may improve patients’ disease-free survival.

## 2. Results

### 2.1. Metastatic Properties of TNBC Cells Are Diminished upon MEF2C’s Silencing

#### 2.1.1. MEF2C’s Expression in TNBC Cells Is Reduced upon Silencing without Compromising Cells’ Viability

**Figure 1:**
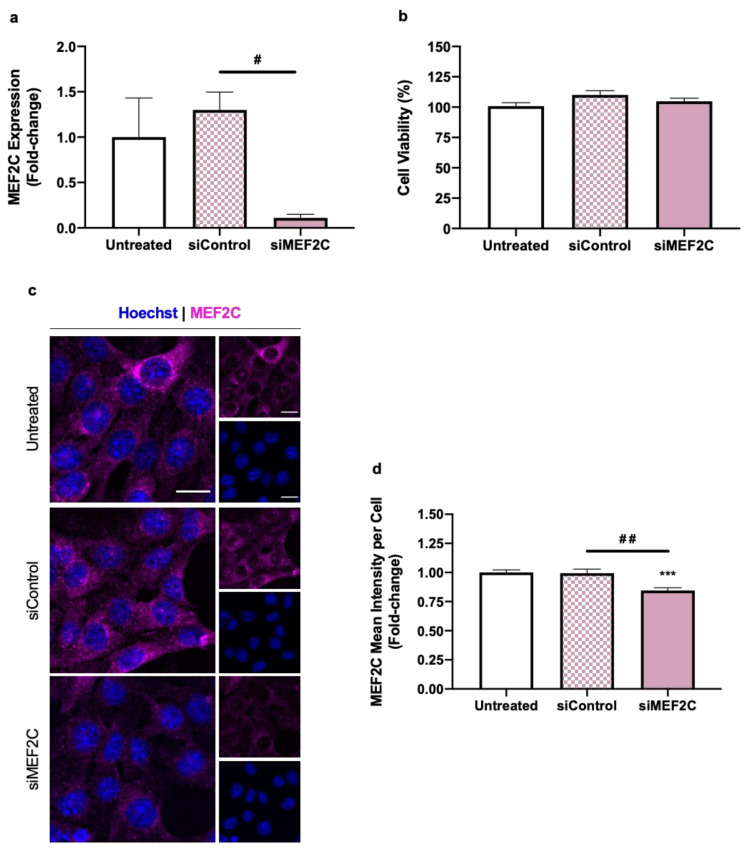
Silencing of myocyte enhancer factor 2C (MEF2C) expression does not compromise triple negative breast cancer (TNBC) cells’ viability. Here, 4T1 cells were incubated with either no addition (untreated) or with siRNAs (siControl and siMEF2C), for 24 h, after which MEF2C’s expression was assessed by real-time quantitative polymerase chain reaction (RT-qPCR) and immunofluorescence (IF), and TNBC cells’ viability was determined by MTT assay. (**a**) RT-qPCR analysis revealed that siMEF2C induced a decrease in MEF2C mRNA expression levels. (**b**) MTT assay showed that the transfection process elicited no toxicity to 4T1 cells. (**c**) IF analysis of MEF2C (magenta) showed that transfection with siMEF2C induced the downregulation of MEF2C protein expression. Nuclei were counterstained with Hoechst 33342 (blue). Scale bar: 20 µm. (**d**) Semi-quantitative analysis of MEF2C’s mean intensity per cell confirmed the decrease in the protein expression. Results are the mean ± SEM (*n* = 3, performed in triplicate for RT-qPCR and MTT) and presented as fold-change (RT-qPCR and IF) or percentage (MTT) vs. untreated cells. Statistical differences are denoted as *** *p* < 0.001 vs. untreated, and by ^#^ *p* < 0.05 and ^##^ *p* < 0.01 between indicated conditions, determined by one-way ANOVA.

To disclose the role of MEF2C in TNBC, a mechanistic study using siRNA targeted to MEF2C (siMEF2C) was conducted in the triple negative 4T1 cell line. The transfection efficiency was assessed by real-time quantitative polymerase chain reaction (RT-qPCR) and immunofluorescence (IF) analysis of MEF2C, ensuring the maintenance of cell viability, via the thiazolyl blue tetrazolium (MTT) assay (Figure 1).

MEF2C’s mRNA levels, appraised by RT-qPCR, were significantly reduced using the siMEF2C (10 nM), as expected, in comparison to siControl-treated (10 nM) cells (Figure 1a). The safety of this transfection in 4T1 cells was then assessed, showing that the siRNAs had no effect on TNBC cells’ viability (Figure 1b). Additionally, the alterations observed in MEF2C’s mRNA levels were validated regarding protein expression within the cells, where MEF2C-silenced cells presented a significant reduction in this protein’s intensity (Figure 1c). In line with these results, the semi-quantitative analysis of IF data showed that the silencing significantly reduced MEF2C’s mean intensity per cell (Figure 1d).

#### 2.1.2. MEF2C Silencing Promotes a Partial EMT in TNBC Cells

**Figure 2:**
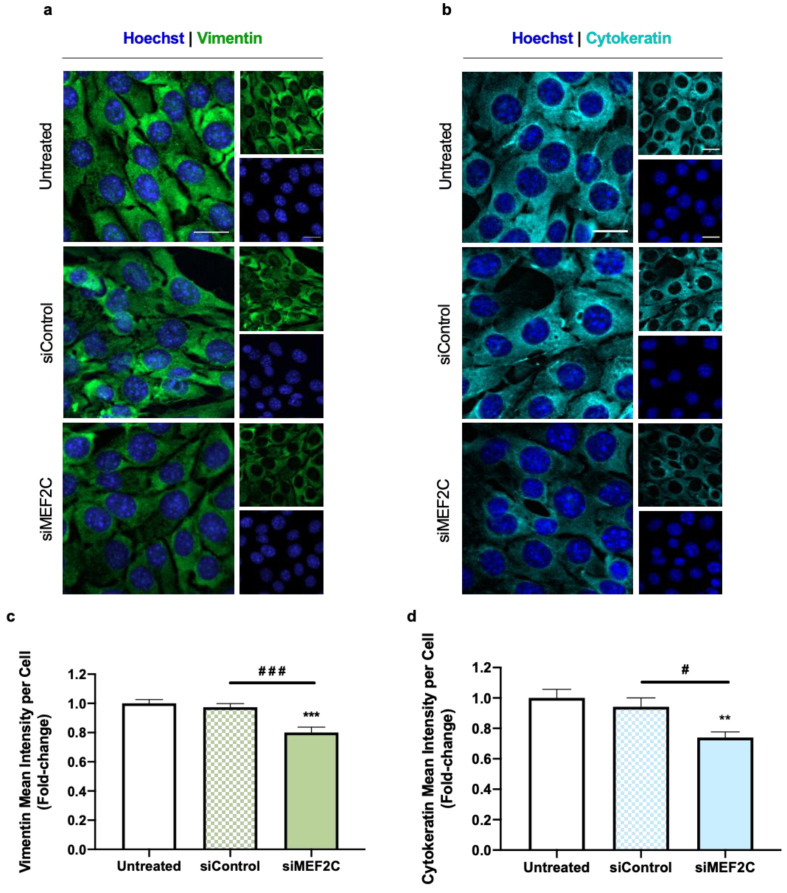
Myocyte enhancer factor 2C (MEF2C) silencing leads to loss of mesenchymal and epithelial properties of triple negative breast cancer (TNBC) cells. Here, 4T1 cells were incubated with either no addition (untreated) or with siRNAs (siControl or siMEF2C), for 24 h. Immunofluorescence analysis of (**a**) the mesenchymal marker vimentin (green) and of (**b**) the epithelial marker cytokeratin (cyan) showed that transfection with siMEF2C induced a decrease in both markers. Nuclei were counterstained with Hoechst 33342 (blue). Scale bar: 20 µm. Semi-quantitative analysis further corroborated (**c**) the decrease in vimentin’s mean intensity per cell, as well as in (**d**) cytokeratin’s mean intensity per cell. Data are given as mean ± SEM (*n* = 3, 10 fields/condition) and expressed as fold-changed from untreated. Significant differences were represented by ** *p* < 0.01 and *** *p* < 0.001 vs. untreated, ^#^ *p* < 0.05 and ^###^ *p* < 0.001 between the indicated conditions, determined by one-way ANOVA.

It has been described that BC cells undergo phenotypic and morphological alterations associated with the occurrence of EMT, in which they acquire a mesenchymal phenotype, becoming more elongated, with an increase in mesenchymal markers like vimentin and the loss of specific epithelial cell markers, such as the intermediate filament cytokeratin [3,30]. These alterations have been associated with the increased invasiveness of tumor cells, which in turn is associated with aggressive behavior [30]. As increased MEF2C expression has been correlated with an aggressive phenotype in brain metastases from TNBC patients [21], we aimed to dissect its role towards EMT. As a way to understand the effects of MEF2C in the mesenchymal and epithelial features of TNBC cells, vimentin and cytokeratin’s expression was assessed by IF analysis (Figure 2). Upon MEF2C’s silencing, there was a decrease in the expression of vimentin (Figure 2a), alongside a decrease in cytokeratin (Figure 2b). The semi-quantitative analysis of IF images showed that cells treated with siMEF2C indeed presented a significant reduction not only in vimentin’s mean intensity per cell (Figure 2c) but also in cytokeratin’s intensity (Figure 2d), as compared with untreated or siControl conditions.

Interestingly, in both stainings, 4T1 cells silenced for MEF2C seemed to present a duality of morphologies, ranging from cuboid to elongated forms. This observation, in addition to the decline seen in both the mesenchymal marker vimentin and in the epithelial marker cytokeratin, points to a partial EMT in TNBC cells silenced for MEF2C.

#### 2.1.3. Loss of MEF2C Promotes a Reduction in TNBC Cells’ Migration

**Figure 3:**
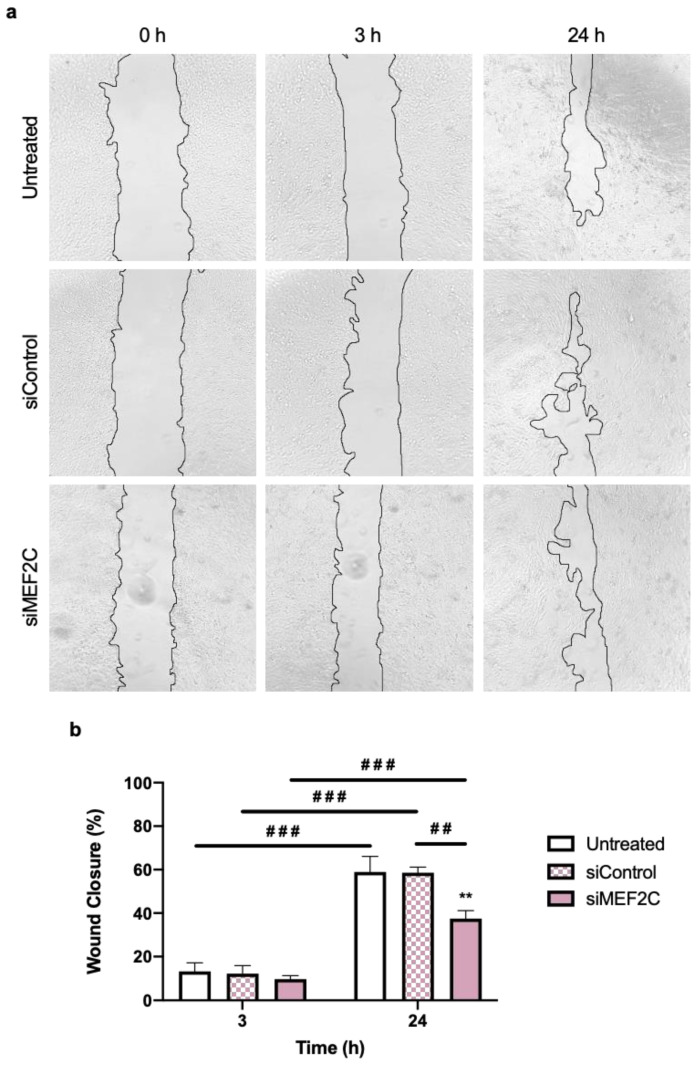
Triple negative breast cancer (TNBC) cells’ migration is reduced in MEF2C-silenced cells. Here, 4T1 cells were incubated with either no addition (untreated) or with siRNAs (siControl and siMEF2C), for 24 h, at which point a wound was inflicted. (**a**) Images of wound closure throughout time (0, 3, and 24 h) were acquired with a phase contrast microscope (100× magnification), showing a strong inhibitory effect on cell migration in cells silenced for MEF2C. This was further confirmed by the semi-quantitative analysis of the (**b**) percentage of wound closure compared to untreated and siControl cells. Data are given as mean ± SEM (*n* = 3, performed in triplicate). Significant differences were represented by ** *p* < 0.01 vs. untreated at 24 h, ^##^ *p* < 0.01 and ^###^ *p* < 0.001 between the indicated conditions, determined by two-way ANOVA test.

In order to perceive the effect of silencing MEF2C on TNBC cells’ invasiveness, the migratory capability of 4T1 cells was determined by a wound-healing assay, by comparison with untreated cells or siControl, after 3 or 24 h of transfection (Figure 3). Although the effects after 3 h were still not pronounced, the smaller gap between the edges indicated that BC cells began to migrate (Figure 3a). Moreover, after 24 h, the silencing of MEF2C led to a clear reduction in the migratory behavior of 4T1 cells. In fact, at this timepoint, both controls (untreated and siControl-treated 4T1 cells) showed the complete closure of some wound sites. This contrasts with the reduction in the percentage of wound closure seen in siMEF2C-treated cells, where no wound site was completely closed (Figure 3a).

The semi-quantitative analysis of the percentage of wound closure (Figure 3b) showed no significant differences between conditions at 3 h. Additionally, the clear alterations in BC cells’ migration seen at 24 h were further corroborated, with both the untreated 4T1 cells and the siControl transfected cells exhibiting a percentage of wound closure of around 59%, whereas MEF2C-silenced cells had the lowest closure of approximately 37%. This translates into a reduction in migration of around 20% between 4T1 untreated and transfected, pointing to a reduction in the migratory phenotype in MEF2C-silenced 4T1 cells.

In sum, 4T1 cells’ migratory capability is reduced by the downregulation of MEF2C, in line with the decreased expression of the mesenchymal marker vimentin. These results suggest an overall contribution of MEF2C to TNBC cells’ invasiveness/aggressiveness, which can be counteracted by the silencing of the protein.

### 2.2. Metastatic Profile of TNBC Cells Is Reduced upon miR-194-5p Overexpression

Previous studies from our team showed the downregulation of miR-194-5p prior to BCBM development, suggesting that this miRNA can act as a tumor suppressor [20,41]. This raised the interest in better comprehending the role of miR-194-5p in TNBC tumorigenesis. To this end, and to increase miR-194-5p’s expression, 4T1 cells were transfected with a pre-miR specific for miR-194-5p or a scramble miR as a negative control.

#### 2.2.1. MiR-194-5p’s Levels in TNBC Cells Are Increased upon Modulation, with Increased Toxicity

**Figure 4:**
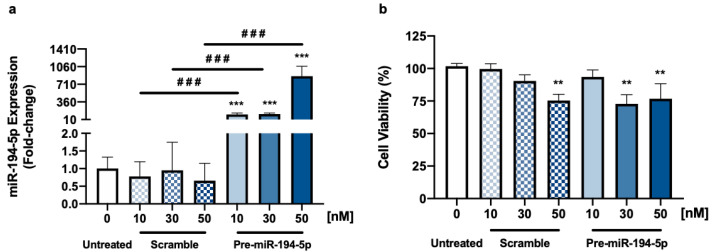
Pre-miR-194-5p at 10 nM increases miR-194-5p’s expression without compromising triple negative breast cancer (TNBC) cells’ viability. Here, 4T1 cells were incubated with either no addition (untreated) or with pre-miR-194-5p or scramble, at 10, 30, and 50 nM, for 48 h. Upon this incubation, cells’ expression levels and viability were analyzed. (**a**) Quantitative analysis by real-time quantitative polymerase chain reaction (RT-qPCR) revealed that miR-194-5p’s expression was increased after 48 h of pre-miR-194-5p exposure, at all the concentrations. (**b**) The effect of pre-miR-194-5p on TNBC cells’ viability, assessed by MTT assay, showed that the transfection process induced toxicity in TNBC cells for 30 and 50 nM, but not for 10 nM. Results are the mean ± SEM (*n* = 3, performed in triplicate) and expressed as fold-change and percentage vs. untreated cells (RT-qPCR and MTT assay, respectively). Statistical differences are denoted as ** *p* < 0.01, *** *p* < 0.001 vs. untreated, and by ^###^ *p* < 0.001 between indicated conditions, determined by one-way ANOVA.

To assess the transfection efficiency, miRNA-194-5p’s expression levels were evaluated via RT-qPCR, while the safety of the transfection was determined by the analysis of 4T1 cells’ viability by the MTT assay (Figure 4).

In an initial screening, different concentrations (10, 30, and 50 nM) of scramble and pre-miR-194-5p were tested to evaluate the effect that each concentration would have on miR-194-5p’s levels. Notably, all the tested concentrations significantly increased this miRNA’s expression, with the highest concentration (50 nM) inducing the greatest upregulation (Figure 4a).

As all the concentrations tested significantly increased miR-194-5p’s levels, the safety of these concentrations for 4T1 cells’ viability was ensured. At 50 nM, both the scramble and the pre-miR-194-5p significantly compromised 4T1 cells’ viability (Figure 4b), which resulted in the exclusion of this concentration from further experiments. Similarly, the 30 nM concentration was excluded since it also induced a significant reduction in 4T1 cells’ viability. Then, the 10 nM concentration was chosen for the subsequent studies, since it did not significantly affect the viability of these cells, while increasing miR-194-5p expression similarly to 30 nM.

#### 2.2.2. EMT in TNBC Cells Is Affected by Increased Levels of miR-194-5p

**Figure 5:**
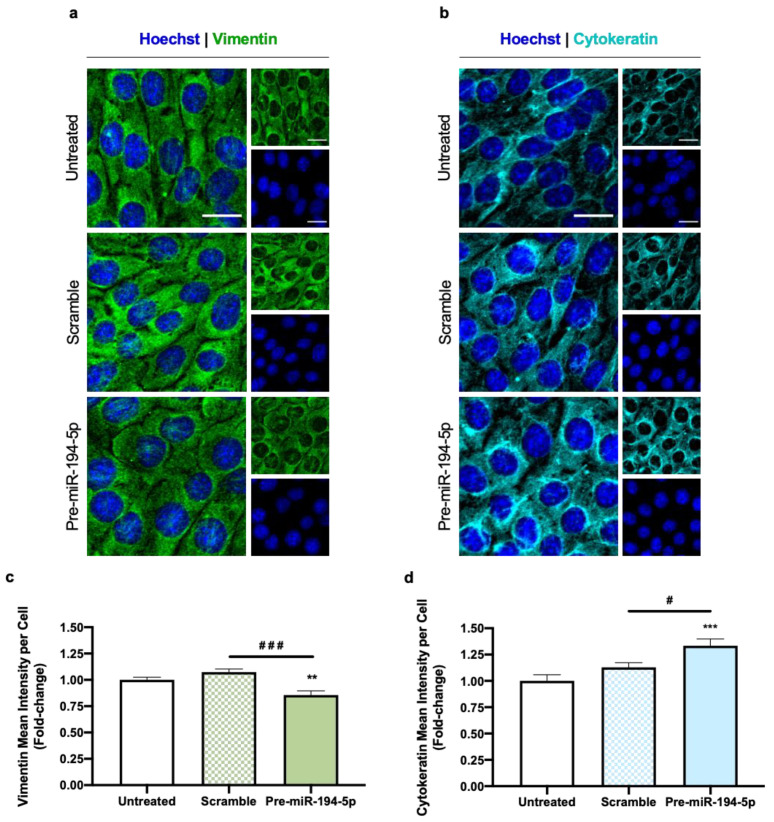
Increase in miR-194-5p levels leads to loss of mesenchymal properties and gain of epithelial features in triple negative breast cancer (TNBC) cells. Here, 4T1 cells were incubated with either no addition (untreated) or with pre-miR-194-5p or scramble, for 48 h. Immunofluorescence analysis of (**a**) the mesenchymal marker vimentin (green) and of (**b**) the epithelial marker cytokeratin (cyan) showed that transfection with pre-miR-194-5p promoted a decrease in vimentin and an increase in cytokeratin. Nuclei were counterstained with Hoechst 33342 (blue). Scale bar: 20 µm. Semi-quantitative analyses further corroborated (**c**) the decrease in vimentin’s mean intensity per cell and (**d**) the increase in cytokeratin’s mean intensity per cell upon pre-miR-194-5p treatment. Data are given as mean ± SEM (*n* = 3, 10 fields/condition) and expressed as fold-change from untreated. Significant differences were represented by ** *p* < 0.01 and *** *p* < 0.001 vs. untreated, ^#^ *p* < 0.05 and ^###^ *p* < 0.001 between the indicated conditions, determined by one-way ANOVA.

As mentioned above, the EMT-associated phenotypic and morphological alterations seen in BC cells are known to be linked with the increased invasiveness of tumor cells, which in turn is associated with aggressive behavior [3,30]. To perceive the putative effects of miR-194-5p on the mesenchymal and epithelial features of TNBC cells, vimentin and cytokeratin’s expression was assessed by IF (Figure 5). All 4T1 cells expressed the mesenchymal marker vimentin, both in untreated and scramble-transfected cells, with a reduction in pre-miR-194-5p-transfected cells (Figure 5a). In contrast, cytokeratin expression, which was also evident in all conditions, was increased when miR-194-5p’s expression was augmented (Figure 5b). The semi-quantitative analysis of IF data corroborated the qualitative observations, since 4T1 cells treated with pre-miR-194-5p presented a significant reduction in vimentin intensity (Figure 5c), concomitant with a significant increase in cytokeratin intensity (Figure 5d).

Collectively, these data point to the modulation of EMT in TNBC cells by pre-miR-194-5p, as the increase in miR-194-5p’s levels not only promoted a reduction in the mesenchymal marker vimentin, but also induced an increase in the epithelial one cytokeratin, in line with reduced TNBC cell invasiveness.

#### 2.2.3. Increased Levels of miR-194-5p Reduce TNBC Cells’ Migration

It is known that when cancer cells undergo morphological alterations associated with EMT, they become more migratory. This ability to invade the surrounding tissue and enter into circulation is a hallmark of metastasis formation [42]. Hence, migratory studies represent a means to appraise cancer cells’ aggressiveness, mirroring an invasive capability.

**Figure 6:**
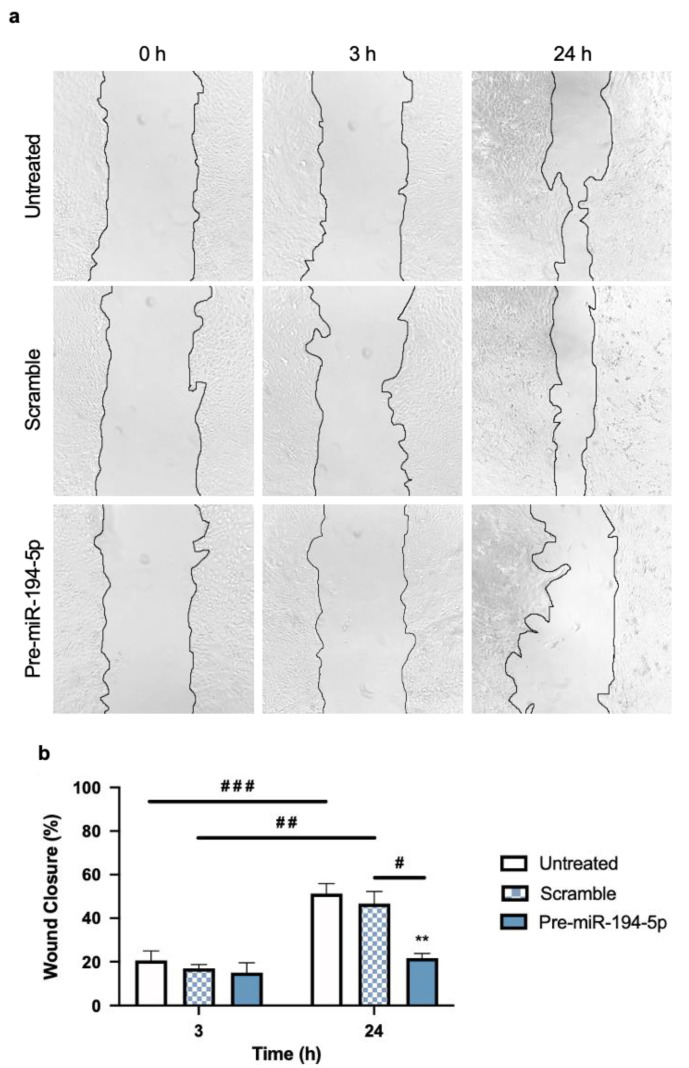
Increase in miR-194-5p levels significantly inhibits triple negative breast cancer (TNBC) cells’ migration. Here, 4T1 cells were incubated with either no addition (untreated) or with pre-miR-194-5p or scramble, for 48 h, at which point 4T1 cells were wounded. (**a**) Images of wound closure throughout time (0, 3, and 24 h) were acquired with a phase contrast microscope (100× magnification) and revealed a strong inhibitory effect on cell migration in cells overexpressing miR-194-5p, validated by the semi-quantitative analysis of the (**b**) percentage of wound closure compared to untreated cells. Data are given as mean ± SEM (*n* = 3, performed in triplicate). Significant differences were represented by ** *p* < 0.01 vs. untreated at 24 h, ^#^ *p* < 0.05, ^##^ *p* < 0.01, and ^###^ *p* < 0.001 between the indicated conditions, determined by two-way ANOVA test.

To assess whether the loss of mesenchymal features and gain of epithelial ones reduces TNBC cells’ migratory capability, a wound-healing assay was performed (Figure 6). Upon 48 h of transfection (with the previously selected 10 nM concentration), the effect of miR-194-5p’s overexpression on 4T1 cells’ migration was assessed. The results obtained showed that, at 3 h, no significant changes in the migration pattern occurred. In contrast, after 24 h, migration was strongly reduced in TNBC cells overexpressing miR-194-5p (Figure 6a). Indeed, at 24 h, both controls (untreated 4T1 cells and scramble) showed an evident increase in wound closure, whereas in cells treated with pre-miR-194-5p the wound remained essentially unclosed.

The semi-quantitative analysis of the percentage of wound closure (Figure 6b) showed no significant differences between conditions 3 h after the end of the transfection period. Additionally, the perceivable visual effect seen at 24 h was further corroborated, with the untreated 4T1 cells presenting a percentage of wound closure (52%) similar to that of cells transfected with the scramble (47%), whereas cells overexpressing miR-194-5p had the lowest closure percentage (22%). This translates into a reduction in migration of around 30% between 4T1 untreated and pre-miR-194-5p transfected cells and highlights the reduction in wound closure promoted by increased levels of miR-194-5p.

Together, these results show that an increase in miR-194-5p’s levels is able not only to reverse EMT in 4T1 cells but also to reduce their migratory capacity, overall contributing to a decrease in TNBC cells’ invasiveness and aggressiveness.

## 3. Discussion

**Figure 7:**
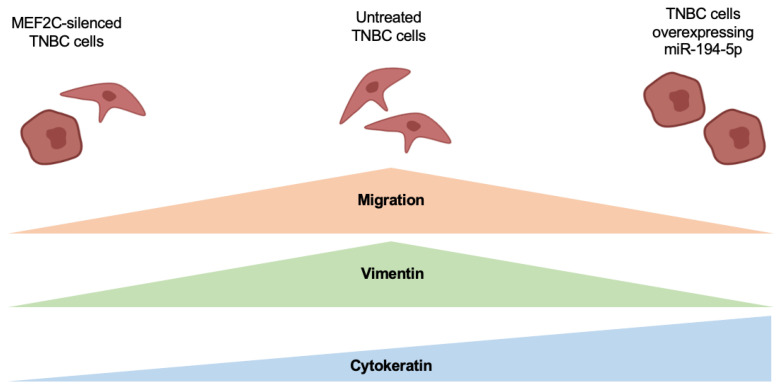
Myocyte enhancer factor 2C (MEF2C) and microRNA (miR)-194-5p modulate triple negative breast cancer (TNBC) cells’ phenotype and migratory capacity. TNBC cells silenced for MEF2C presented a decrease in migratory capability, along with reduced vimentin and cytokeratin levels, suggestive of a partial epithelial–mesenchymal transition (EMT). TNBC cells overexpressing miR-194-5p also presented a decline in migration and vimentin expression, whereas cytokeratin expression was boosted, concomitant with a reduction in TNBC cells’ aggressiveness.

Previous observations from our group pointed to miR-194-5p and to MEF2C as new players in TNBC tumorigenesis [20,21,41]. The present work goes further in our understanding of MEF2C’s and miR-194-5p’s roles in tumorigenesis, by modulating the expression of such emerging players and demonstrating the impact on TNBC cells’ aggressiveness mechanisms, such as EMT and invasiveness, as outlined in Figure 7.

EMT is one of the most studied tumorigenic processes and yet, in some cases, like TNBC, the mechanisms preceding this phenomenon are still poorly understood [43]. During EMT, tumor cells undergo a series of intricate modifications, namely in cytoskeletal organization with the loss of cell-to-cell connections, in which they partially or completely lose their epithelial phenotype and acquire mesenchymal characteristics [42,44]. Such characteristics allow tumor cells to present plasticity and survive under changes in environment. For instance, in BCBM formation in vivo, we have observed that vimentin expression in TNBC appears to be essential for tumor cell extravasation and brain colonization, a phenomenon observed at very early timepoints [45]. In this work, we reveal that TNBC cells silenced for MEF2C present a reduction not only in the mesenchymal marker vimentin but also in the epithelial marker cytokeratin, suggesting that MEF2C silencing induces a partial EMT in TNBC cells. In the literature, a partial EMT is considered a “metastable” intermediate transition, characterized by weak cell–cell junctions, in addition to increased motility and invasive properties [46]. This process has been observed in different cancer types, including head and neck [46], lung [47], renal [48], and BC [49,50], reinforcing the present findings.

Our results regarding TNBC cells’ migration showed a reduced migratory capability in MEF2C-silenced cells, when compared to the controls. This was particularly visible at 24 h, where not even 50% of the wound was closed, suggesting that MEF2C downregulation significantly limits TNBC cells’ motility and invasive phenotype. It is known that a key step in metastatic development is the ability of tumor cells to invade the surrounding tissue [51] and, to our knowledge, MEF2C has not been directly related to the migratory capability of TNBC cells. In fact, MEF2C’s dysregulation has been correlated with different diseases, including several types of cancer [52]. In accordance with our data, MEF2C’s silencing was shown to promote a reduction in glioma cells’ migration and proliferation, in addition to an increase in cell apoptosis [53]. Similarly, in hepatocellular carcinoma, MEF2C-silenced cells appeared to counteract the migratory effect caused by vascular endothelial growth factor (VEGF) overexpression, by maintaining the percentage of the wound area [16]. On the other hand, in cervical cancer, several tumorigenic processes, like proliferation, migration, and invasion, were inhibited when MEF2C’s expression levels were upregulated [54]. Indeed, in gastric cancer cells, the opposite effects were observed with the knockdown of MEF2C, which led to enhanced migration [55]. Therefore, MEF2C appears to have a dual effect on cancer cells’ migration. Nonetheless, our results evidence, for the first time, that MEF2C silencing leads to a significant reduction in TNBC cells’ migration, corroborating its tumorigenic role.

The dysregulation of miRNAs can affect EMT through interaction with a variety of specific pathways and protein targets, which, in turn, will affect several pathogenic processes [56,57]. In line with this, the effects of the overexpression of miR-194-5p on EMT features were assessed by the analysis of a mesenchymal and an epithelial marker. Our results showed a substantial reduction in vimentin, in opposition to an increase in cytokeratin, by augmented levels of the miRNA. This observation discloses the modulation of EMT in TNBC cells through augmented levels of miR-194-5p, which can reflect a reduction in TNBC cells’ invasiveness and points to the tumor suppressor role of this miRNA. A tumor suppressor role of miR-194-5p has also been observed in several types of cancer, such as Wilms tumors [58], colorectal adenocarcinoma [59,60], glioma [61], and gastric cancer [62]. Similarly to our results, the induced overexpression of miR-194-5p led to a decline in mesenchymal markers like vimentin, neuronal (N)-cadherin, and Twist1/2, as well as to an increase in epithelial markers, such as epithelial (E)-cadherin, ultimately suggesting the total inhibition of EMT [58,59,60,61,62]. Interestingly, in BC, miR-194-5p knockdown was shown to suppress tumor growth, yet the effect of this dysregulation on EMT has not been established [36].

To our knowledge, a direct relation between miR-194-5p’s dysregulation and TNBC cells’ migratory capability remains to be determined. Bearing this in mind, and to better understand the impact of overexpressing miR-194-5p, a wound-healing assay was performed, where it was possible to observe that TNBC cells overexpressing miR-194-5p had slower closure than the respective controls, particularly at 24 h. At this timepoint, TNBC cells’ migration was strongly reduced with the pre-miR-194-5p, indicating that increased levels of this miR significantly limit TNBC cells’ motility and invasive phenotype. Depending on the cancer type or subtype, miR-194-5p can have dual behavior, acting as an oncogene or tumor suppressor. In fact, miR-194-5p was found to be upregulated in BC tissues and its knockdown led to an inhibitory effect in the same tumorigenic processes of BC cells [36]. These data are in line with another study, in which miR-194-5p’s overexpression in BC cells was associated with aggressive behavior, by counteracting a specific long non-coding RNA—LNC00641—and increasing tumorigenic parameters, such as migration and invasion [34]. In contrast, a tumor suppressor role has been seen in several types of cancer, such as in non-small-cell lung cancer [40], osteosarcoma [63], gastric cancer [64], and colorectal cancer [65], where miR-194 had an inhibitory effect on tumorigenic processes such as migration, proliferation, and invasion. Hence, the different roles of miR-194-5p emphasize the dual effect that this miRNA may have, depending on the cell and cancer types. In our study, this miRNA appears to have a tumor suppressor role in TNBC, since its overexpression is aligned with a decrease in the migratory capability of BC cells and, thus, with a putative reduction in aggressiveness, as corroborated by a reduction in BC cells’ mesenchymal phenotype.

The inhibitory effect on EMT caused by the overexpression of miR-194-5p in TNBC cells seems to show great potential towards the inhibition of invasive properties. The seeming duality of miR-194-5p is an incredibly rich and valuable topic worth investigating further as it may hold the key to the diagnosis, prevention, and treatment of many different types of cancer. This study reports, for the first time, an inhibitory effect of not only MEF2C but also miR-194-5p in TNBC cells’ migratory and invasive capacity, bringing a new understanding of these two players in the pathogenesis of TNBC. Notwithstanding, the preliminary safety screening also provided a basis for a possible therapeutic approach to tackle TNBC, since only pre-miR-194-5p at 30 nM and not the scramble showed some toxicity against TNBC cells. For this reason, this concentration of pre-miR-194-5p could be further studied in a delivery system to specifically target BC cells. The use of nanoparticle deliveries of miRNAs has already been studied in TNBC, tackling different stages of tumor development and metastasization. For instance, the suppression of TNBC migration was successfully achieved with the encapsulation of miR-203, whereas tumor growth and metastasis formation were inhibited and delayed using the nanoparticle delivery of both miR-34a and miR-10b [66,67]. Additionally, a combined therapy consisting of the encapsulation of both orlistat and antisense-miR-21 led to a better cellular therapeutic response [68]. Together with cluster of differentiation (CD)133, a highly expressed marker in TNBC, miR-21 was also used in a mouse model, showing significant tumor growth inhibition [69]. These promising studies emphasize the unexplored interest in understanding the effect of the specific targeted delivery of pre-miR-194-5p at a concentration of 30 nM, as a way to limit TNBC cells’ survival.

## 4. Materials and Methods

### 4.1. Cell Culture Conditions

A murine mammary carcinoma triple negative 4T1 cell line (ATCC, Middlesex, UK) was used. The 4T1 cells were cultured and maintained in Roswell Park Memorial Institute (RPMI) 1640 (Sigma-Aldrich, St. Louis, MO, USA) supplemented with 2 mM l-glutamine (Sigma-Aldrich) and 5% (*v*/*v*) fetal bovine serum (FBS, Sigma-Aldrich), at 37 °C, in a humid atmosphere enriched with 5% CO_2_, as is typical in our lab [70].

### 4.2. Transient Transfection

In vitro transfection studies in 4T1 cells used a small interfering RNA (siRNA) specific to MEF2C (siMEF2C) and its respective negative control (siControl), purchased from Ambion (Life Technologies, Carlsbad, CA, USA). Pre-miR-194-5p and miRNA negative control (scramble) were purchased from Ambion. The 4T1 cells were seeded, and, after 24 h, they were transiently transfected with the siRNAs (siMEF2C or siControl, 10 nM each) or pre-miR-194-5p (10, 30 and 50 nM), or scramble, using Lipofectamine™ 3000 (Invitrogen, Thermo Fisher Scientific, Waltham, MA, USA) and Opti-MEM™ Reduced Serum Medium (Gibco, Thermo Fisher Scientific). Control assays with Opti-MEM™ Reduced Serum Medium alone (untreated) were run in parallel. Transfection mixes were diluted in complete cell medium, and transfections were stopped after 24 h of siRNA incubation or 48 h of pre-miR-194-5p/scramble incubation according to the manufacturer’s instructions.

### 4.3. Quantitative Real-Time Polymerase Chain Reaction (RT-qPCR)

Total RNA was isolated from cells using TRIzol reagent (Grisp, Porto, Portugal), according to the manufacturer’s instructions. For gene expression, RNA was then transcribed into cDNA, using the Xpert cDNA Synthesis Kit (Grisp), while for miRNA expression the miRCURY Locked Nucleic Acid (LNA) RT Kit (Qiagen, Hilden, Germany) was used, according to the manufacturer’s instructions. In the latter, prior to the reverse transcription reaction, the synthetic RNA UniSp6 RNA spike-in (Qiagen) was added to the mixture. The reactions were performed on a Biometra T-Combi thermocycler (Analytic Jena, Jena, Germany), using the following conditions for gene expression: 65 °C for 5 min, 50 °C for 15 min, and 85 °C for 5 min to heat-inactivate the reverse transcriptase, and cooling down and storage at 4 °C. Meanwhile, for miRNA expression the following conditions were used: 42 °C for 60 min, 95 °C for 5 min to heat-inactivate the reverse transcriptase, and cooling down and storage at 4 °C.

RT-qPCR was performed using the QuantStudio™ 7 Flex Real-Time PCR System (Applied Biosystems, Thermo Fisher Scientific) [41]. The Xpert Fast SYBR Green (Grisp) was used for gene expression, according to the manufacturer’s instructions, diluted at 1:2 and using the following conditions: 1 cycle of 95 °C for 2 min, 40 cycles of 95 °C for 5 s, and 60–65 °C for 20–30 s, followed by a dissociation/melting curve analysis. In the case of miRNA expression, the miRCURY LNA SYBR Green PCR Kit (Qiagen) was used according to the manufacturer’s instructions, using cDNA diluted at 1:6. The following conditions were used: 50 cycles of 95 °C for 15 s, 56 °C for 30 s, 72 °C for 30 s, and a ramp rate of 1.6 °C/s, followed by a melting curve analysis.

Forward and reverse primer pairs for MEF2C and housekeeping gene glyceraldehyde 3-phosphate dehydrogenase (GAPDH) (Table 1) were purchased from Stab Vida (Caparica, Portugal), while specific predesigned LNA primer pairs for miR-194-5p (mmu-miR-194-5p) and endogenous control miR-16-5p (mmu-miR-16-5p) were purchased from Qiagen.

RT-qPCR was performed in 384-well plates, with each sample examined in triplicate for three independent experiments, and a no-template control was included for each sample amplification. Determination of the threshold cycle was performed using the QuantStudio^TM^ 7 Flex Real-time PCR software (Applied Biosystems), and the quantifications performed by the ∆∆Ct method. The results are presented as fold-changes.

### 4.4. Cell Viability Assay

Upon transfection and to assess the effect of this procedure in TNBC cells, 4T1 cells’ viability was evaluated using the MTT assay, adapted from a previous description [71]. The 4T1 cells were seeded into uncoated 96-well plates using a volume of 200 μL at a density of 2.5 × 10^4^ cells/mL, and, after the transfection period (24 h for siRNA and 48 h for pre-miR), the medium was discarded and 0.5 mg/mL of MTT (Alfa Aesar, Haverhill, MA, USA) diluted in RPMI was added to each well. Then, cells were incubated for 1 h at 37 °C and 5% CO_2_. Supernatants were removed and the formazan crystals were solubilized with a solution of 0.04 N HCl in isopropanol (Honeywell, Charlotte, NC, EUA). Absorbance values were obtained using a microplate reader (Zenyth 3100, Anthos Labtec Instruments, Salzburg, Austria) at 595 nm and results are presented as a percentage of untreated 4T1 cells. All experiments were performed in triplicate for three independent experiments.

### 4.5. Immunofluorescence

MEF2C’s levels and phenotypic alterations in tumor cells upon selected transfections were evaluated by IF [70] analysis of the transcription factor MEF2C and the epithelial and mesenchymal markers (cytokeratin and vimentin, respectively). Briefly, 4T1 cells were seeded onto coverslips in uncoated 24-well plates using a volume of 500 μL at a density of 5 × 10^4^ cells/mL and fixed post-transfection with freshly prepared 4% (*v/v*) paraformaldehyde (PFA, Sigma-Aldrich) in phosphate-buffered saline (PBS) for 20 min at room temperature. Following fixation, cells were washed with PBS and permeabilized for 5 min with 0.3% Triton X-100 (VWR International, Radnor, PA, USA), blocked for 60 min at room temperature with 3% bovine serum albumin (BSA, Sigma-Aldrich), and incubated overnight at 4 °C with the primary antibodies, followed by incubation with the corresponding secondary antibodies for 60 min at room temperature. Both primary and secondary antibodies (Table 2) were diluted in blocking solution. Nuclei were counterstained with Hoechst 33342 dye (Thermo Fisher Scientific, 20 μM) for 10 min at room temperature. Between incubations, cells were washed three times with PBS. Methanol (Honeywell)-dehydrated cells were then mounted in microscopy slides with dibutylphthalate polystyrene xylene (DPX (Merck Millipore, Burlington, MA, EUA)), properly dried, and stored at 4 °C until image acquisition. Negative control assays were performed without a primary antibody. For each protein, three independent experiments were performed.

### 4.6. Wound-Healing Assay

The migratory behavior of tumor cells silenced for MEF2C or overexpressing miR-194-5p was evaluated through a wound-healing assay [71]. Briefly, 4T1 cells were seeded in uncoated 96-well plates using a volume of 200 μL at a density of 5 × 10^4^ cells/mL (siRNA) or 4 × 10^4^ cells/mL (pre-miRNA), and, after the transfection period, a sterile 10 μL pipette tip was used to scrape a longitudinal wound through the cell monolayer, with an even diameter. Cells were washed with Hank’s Balanced Salt Solution (HBSS, Gibco), followed by 24 h incubation in RPMI, after which BC cells’ migration and wound closure were monitored over time (0, 3, and 24 h). Three independent experiments were performed in triplicate. The results are presented as a percentage relative to untreated cells at the corresponding timepoint.

### 4.7. Image Acquisition

Images from 10 fields per coverslip (*n* = 3) were acquired at the Faculty of Sciences, University of Lisbon, BioIsi Facility. For IF, images were examined using an Olympus BX60 microscope equipped with an Olympus U-RFL-T Mercury lamp and Hamamatsu Orca R2 cooled monochromatic CCD camera, using a 40× oil objective.

For the wound-healing assay, images were acquired at 0, 3, and 24 h after the wound was inflicted (*n* = 3, performed in triplicate, one image per well), using a 10× objective with a phase contrast microscope (Carl Zeiss, Primovert, NY, USA).

### 4.8. Image Analysis

IF images were analyzed using the ImageJ version 1.53 (National Institutes of Health, Bethesda, MD, USA) software. Immunolabelling was quantified by measuring the mean intensity per image, divided by the number of cells in the same image. In the wound-healing assay, wound closure was quantified using the freehand selection tool in the ImageJ software to measure the area between the wound’s ends. The results are presented as the wound closure percentage of the control (0 h), using the following Equation (1):

(1) $$\%\;closure=100-\frac{Area\;at\;chosen\;timepoint\times 100}{Area\;at\;0\;h}$$

### 4.9. Statistical Analysis

Results were analyzed with GraphPad Prism^®^ 8.4.3 (GraphPad Software, San Diego, CA, USA) and are expressed as the mean ± SEM, representing the average from three independent experiments (*n* = 3). Results’ normality was tested with the D’Agostino–Pearson test. When normality was verified, the significance of differences between treatments and controls was evaluated by analysis of variance with the one-way ANOVA or two-way ANOVA multiple comparison test, in the case of grouped data, such as in the wound-healing assay. Differences were considered statistically significant when *p* < 0.05.

## 5. Conclusions

In the present study, miR-194-5p and MEF2C’s roles have been highlighted in key mechanisms involved in TNBC cells’ aggressiveness, such as their invasion and migration, owing to morphological and phenotypic alterations. These effects were observed for the first time in TNBC, revealing miR-194-5p and MEF2C as new players in the tumorigenic processes of BC. In sum, this study offers a new understanding of the roles and contributions of MEF2C and miR-194-5p in tumorigenesis, by providing new information beyond the current state of the art in BC and, consequently, disclosing novel modulation targets aimed to improve patients’ survival, particularly those presenting brain metastases.

## Figures and Tables

**Table 1:** Real-time quantitative PCR primers.

Target	Primer Sequence	Length (Base Pairs)
GAPDH	Forward: 5-GTG GCA AAG TGG AGA TTG TTG CC-3	23
	Reverse: 5-GAT GAT GAC CCG TTT GGC TCC-3	21
MEF2C	Forward: 5-AGA TCT GAC ATC CGG TGC AG-3	20
	Reverse: 5-TCT TGT TCA GGT TAC CAG GT-3	20

GAPDH, glyceraldehyde-3-phosphate dehydrogenase; MEF2C, myocyte enhancer factor 2C.

**Table 2:** Summary of the experimental conditions for immunofluorescence analysis.

Target Protein	Primary Antibody	Secondary Antibody
MEF2C	MEF2C (1:100) Santa Cruz Biotechnology, #sc-518152, Mouse	Alexa Fluor 555 (1:500) Thermo Fisher Scientific, #A21428, Goat Anti-Mouse
Cytokeratin	Pan-Cytokeratin (1:50) Thermo Fisher Scientific, #MA512231, Mouse	Alexa Fluor 488 (1:500) Thermo Fisher Scientific, #A11001, Goat Anti-Mouse
Vimentin	Vimentin (1:100) Thermo Fisher Scientific, #MA3745, Mouse	

MEF2C, myocyte enhancer factor 2C.

## Data Availability

All data generated or analyzed during this study are included in this published article.
